# Non-invasive longitudinal imaging of VEGF-induced microvascular alterations in skin wounds

**DOI:** 10.7150/thno.65287

**Published:** 2022-01-01

**Authors:** Yu-Hang Liu, Lorenz M. Brunner, Johannes Rebling, Maya Ben-Yehuda Greenwald, Sabine Werner, Michael Detmar, Daniel Razansky

**Affiliations:** 1 Institute for Biomedical Engineering and Institute of Pharmacology and Toxicology, Faculty of Medicine, University of Zurich, Zurich, Switzerland; 2 Institute for Biomedical Engineering, Department of Information Technology and Electrical Engineering, ETH Zurich, Zurich, Switzerland; 3 Institute of Pharmaceutical Sciences, ETH Zurich, Zurich, Switzerland; 4 Institute of Molecular Health Sciences, Department of Biology, ETH Zurich, Zurich, Switzerland

**Keywords:** Angiogenesis, intravital microscopy, photoacoustic, skin wound, vascular endothelial growth factor

## Abstract

**Background:** Microcirculation is essential for skin homeostasis and repair. A variety of growth factors have been identified as important regulators of wound healing. However, direct observation and longitudinal monitoring of skin remodeling in an unperturbed *in vivo* environment remains challenging.

**Methods:** We report on non-invasive longitudinal imaging of the wound healing process in transgenic mice overexpressing vascular endothelial growth factor A (VEGF-A) in keratinocytes by means of large-scale optoacoustic microscopy (LSOM). This rapid, label-free, high throughput intravital microscopy method averts the use of dorsal skin-fold chambers, allowing for fully non-invasive repeated imaging of intact wounds with capillary resolution over field-of-view spanning several centimeters.

**Results:** We observed VEGF-driven enhancement of dermal vascularization in ears, dorsal skin and healing wounds and quantified the hemoglobin content, fill fraction, vessel diameter and tortuosity. The *in vivo* findings were further corroborated by detailed side-by-side classical histological whole-mount vascular stainings and pan-endothelial CD31 immunofluorescence.

**Conclusion:** The new approach is suitable for supplementing or replacing the cumbersome histological procedures in a broad range of skin regeneration and tissue engineering applications.

## Introduction

Skin is the first line of defense against exogenous insults like allergens, irritants and pathogens 1,2. Once homeostasis is disrupted by injury or malfunction, a large cascade of processes is induced. One major event is angiogenesis, the development of new blood vessels from existing vasculature through sprouting. To this end, a variety of pro-angiogenic factors such as placental growth factor (PLGF), fibroblast growth factor 2 (FGF-2), and vascular endothelial growth factor A (VEGF-A) and VEGF-C have been identified as important regulators of wound angiogenesis 3-5. Among them, VEGF-A promoted angiogenesis and normalized wound healing when applied topically in healing-impaired diabetic mice, which are characterized by insufficient angiogenesis 6. The enhanced vascularization provided more oxygen and nutrients, thereby increasing the proliferative rate of epithelial and stromal cells. VEGF-A further plays an essential role in other pathological conditions such as psoriasis 7 and tumor formation 8.

Since VEGF-A target proteins and specific endothelial markers can be easily identified in tissue sections, histology and immunofluorescence stainings constitute the mainstay approach for studying effects of growth factors that control angiogenesis. However, evaluation of classical histology data is often afflicted with non-standardized procedures for tissue handling, fixation and staining, quantification challenges, and human errors 9. Most importantly, these time-consuming procedures can only be conducted* ex vivo*, thus preventing observations of longitudinal dynamics in the same animal.

Fluorescence microscopy 10-14 has been employed as an alternative tool to study the skin vasculature *in vivo*. However, the minuscule field of view (FOV) 13 and limited penetration dominated by intense photon scattering restrict the use of intravital microscopy for imaging the skin 12. Besides, extrinsic contrast agents are commonly required to identify vascular structures within the skin when employing fluorescence-based methods 14,15. Label-free visualization of skin vascular network can alternatively be done with laser speckle contrast imaging and laser Doppler imaging 16, albeit with poor lateral resolution. In addition, Doppler optical coherence tomography 16, high-speed video capillaroscopy 17, and sidestream dark field imaging 18 could monitor fine vasculatures down to capillary level with blood flow information; nevertheless, their FOVs are typically limited to a few millimeters or less. On the other hand, optoacoustic (OA) microscopy integrates the merits of optics and ultrasound (US), thus providing strong intrinsic optical absorption contrast from blood, high spatial resolution and deep tissue penetration 19,20. Based on absorption spectra of the intrinsic targets of interest, OA microscopes were so far used for a number of applications ranging from cerebrovascular imaging to label-free histology and assessment of psoriatic skin conditions in humans 15,19-21.

Here, we examine the use of optical-resolution, label-free, large-scale optoacoustic microscopy (LSOM) 22 and automated vessel analysis for non-invasive detection and longitudinal observation of VEGF-A-induced vascular alterations in normal and wounded skin. The method averts the surgical implantation of conventional dorsal skin-fold chambers 23, which is replaced by a 3D-printed dorsal skin mount allowing for non-invasive repeated imaging of intact wounds with capillary resolution over extremely large FOV spanning several centimeters. A keratin 14 promoter-driven transgenic mouse model of VEGF-A overexpression (K14-VEGF-A) was studied, owing to its amplified dermal microvasculature compared to wild-type littermates and its effects on wound angiogenesis and repair 7,24-26.

## Results

### Vascularization data from *in vivo* LSOM scans of the mouse ear correlate with classical histology

**Figure 1:**
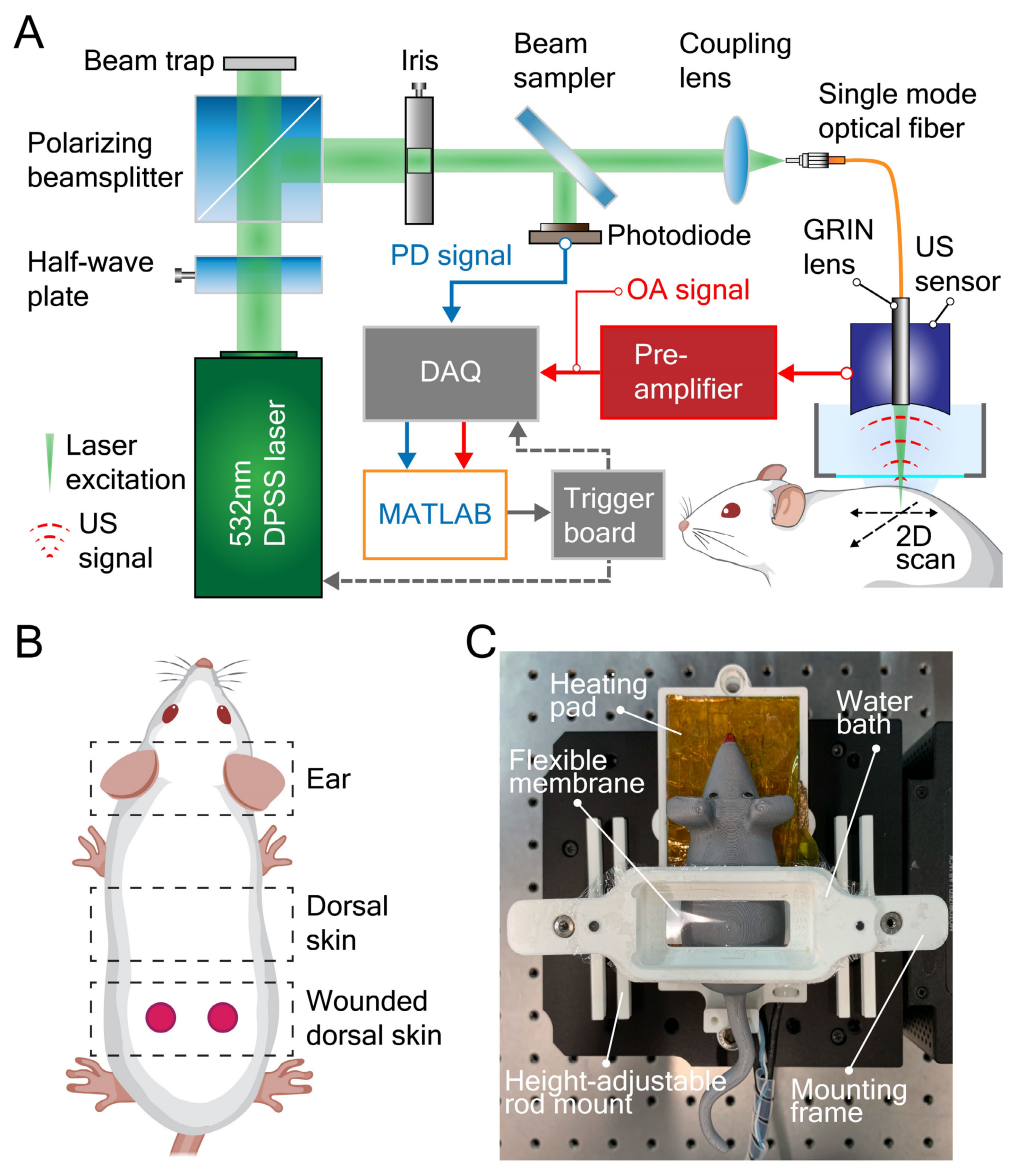
Large-scale optoacoustic microscopy (LSOM) and the customized dorsal imaging mount. (**A**) Schematic representation of the rapid scanning LSOM setup. (**B**) Skin vasculature was evaluated *in vivo* in the ear and in non-wounded and wounded dorsal skin. (**C**) A photograph of the assembled 3D-printed mount. The flexible membrane was fixed between the water bath and the mounting frame, which applies minimal pressure to flatten the dorsal skin while not perturbing the circulation.

**Figure 2:**
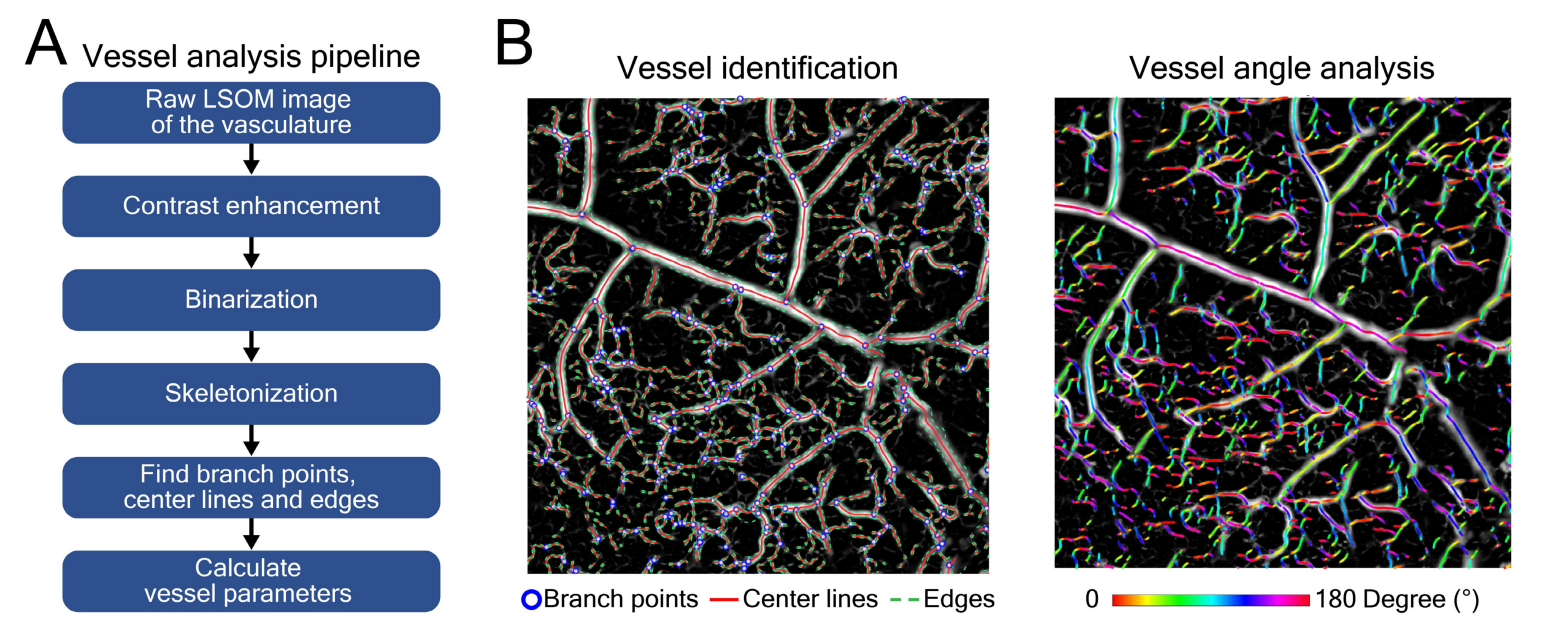
Automatic vessel segmentation and analysis algorithm (AVSA) for skin vasculature assessment. (**A**) Analysis pipeline of the AVSA from raw LSOM images to quantifiable vessel parameters. (**B**) The branch points, center lines and edges of various dorsal skin vessels (from veins to capillaries) of a VEGF-A mouse were identified by the AVSA. As an example, the angles of individual vessels were calculated and labeled with different colors, which were then used to analyze the vessel tortuosity.

The label-free LSOM used for non-invasive imaging of microvascular networks in the mouse ear skin and non-injured/wounded dorsal skin is schematically displayed in Figure 1A-B. Basically, the image contrast at the 532 nm light wavelength used for optoacoustic excitation primarily stems absorption by hemoglobin 27. The volumetric images of the tissue absorption contrast are rendered by sequential scanning of the focused light spot along with the spherical ultrasound detector. A customized 3D-printed skin mount (Figure 1C) was used to place the animals directly under the LSOM for *in vivo* imaging, without any prior invasive surgical procedure 22. After image acquisition, the skin vasculature was analyzed by an automatic vessel segmentation and analysis algorithm (AVSA) to further compare the vascular differences between wild-type (WT) and transgenic VEGF-A mice (Figure 2). Please refer to *Materials and Methods* for more details on the LSOM and AVSA methods.

**Figure 3:**
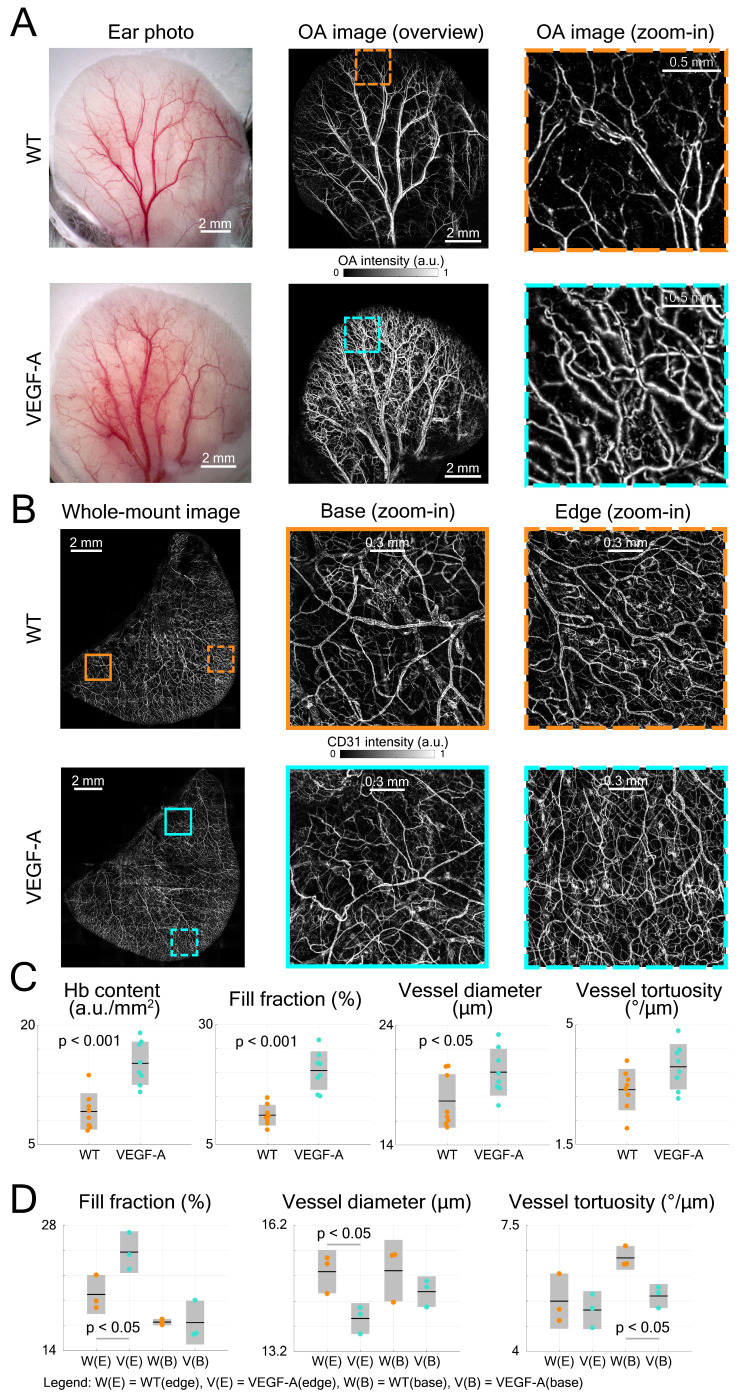
Quantification of mouse ear skin vasculature in WT and VEGF-A mice. (**A**) Color photographs and LSOM images of mouse ear vasculature. The overview LSOM images were consistent with the ear photographs taken by the USB microscope. The zoom-in images further showed different vascularization in the WT and VEGF-A mice (orange and turquoise dashed boxes, respectively) with the latter exhibiting higher vessel density around the edges. (**B**) Whole-mount images of mouse ear skin immunostained for CD31. Whole-mount ear images also demonstrate similar results as the LSOM images with a larger number of microvessels in VEGF-A mice in the edge regions, whereas the vascular networks were similar at ear base regions. (**C**) Multiple parameters of LSOM images were analyzed to evaluate the vasculature-related difference between mice of both genotypes: dots represent median vessel parameters per location; black lines represent mean values; gray boxes represent standard deviation. (**D**) Statistical analysis of different vascular parameters in WT and VEGF-A mouse ears at edge (E) and base (B) regions of whole-mount stainings. Three edge and three base regions (1.7 × 1.7 mm^2^) of each whole-mount ear were used for evaluation.

**Figure 4:**
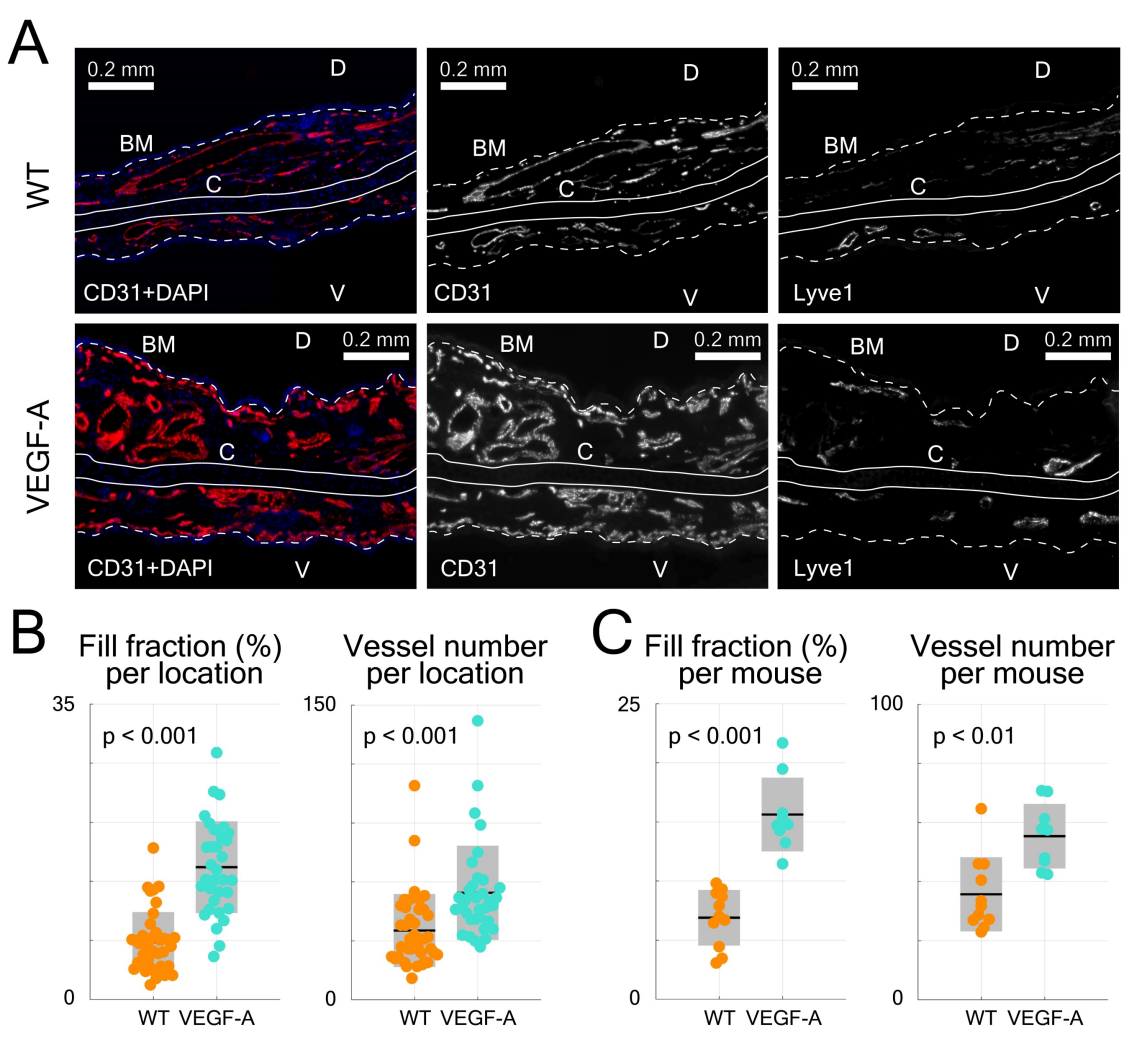
Evaluation of immunofluorescence-stained ear skin sections. (**A**) Representative photomicrographs of CD31/Lyve1-stained ear sections in WT and VEGF-A mice, including images showing stainings with CD31 or Lyve1 antibodies. Nuclei were counterstained with DAPI. Lyve1 staining was used to distinguish blood vessels from lymphatic vessels. White dotted line represents basement membrane (BM), C = cartilage, D = dorsal, V = ventral. (**B**) Quantification of data from different areas of immunostained ear skin sections (tip, tip-middle, middle-base and base). Sections from VEGF-A transgenic mice show a significant increase in fill fraction (%) and vessel number in all analyzed compartments. (**C**) Quantification of data from individual locations averaged per mouse (n = 4 locations per mouse, n = 10 mice). VEGF-A transgenic mice showed a significant increase in fill fraction (%) and vessel number.

The LSOM performance was first evaluated by imaging the mouse ear vasculature. Color photographs of both WT and VEGF-A transgenic mouse ears were captured for comparison, revealing more extensive vascularization in the VEGF-A mouse, especially around the edges (Figure 3A). We then used LSOM to rapidly acquire label-free *in vivo* overview images of the whole ear along with the zoom-ins around the edges (Figure 3A). The mouse ear was placed on a 3D-printed board and gently fixed by a customized thin film holder; thus, no head or ear motion was observed during the image acquisition. The number of microvessels was higher in the VEGF-A mouse ears, which is consistent with the ear photographs and our previous results showing an increased microvascular density in the skin of these animals 24. *Ex vivo* whole-mount ear skin imaging (Figure 3B) and histology (Figure 4A) were also performed to observe the vasculature in WT and VEGF-A mice, with the help of the pan-endothelial marker CD31. Whole-mount ear images showed a higher vascular density around the edges in VEGF-A mice and similar vessel distributions in central regions in mice of both genotypes (Figure 3B).

Four parameters, including Hb content, fill fraction, vessel diameter and tortuosity were analyzed by AVSA in both WT and VEGF-A mice (Figure 3C). The Hb content in VEGF-A mice (mean 15.2 a.u./mm^2^; range: 11.62 - 19.03 a.u./mm^2^) was significantly higher (p << 0.001) than in WT mice (mean 9.17 a.u./mm^2^; range: 6.79 - 13.72 a.u./mm^2^). Similarly, the fill fraction was significantly higher (1.83-fold increase, p << 0.001) in VEGF-A mice (20.40%; range: 15.13 - 26.79%) compared to WT mice (11.13%; range: 8.12 - 14.8%). On the other hand, differences in the vessel diameter (WT: 17.67 µm; VEGF-A: 20.08 µm; p = 0.038) and vessel tortuosity (WT: 3.10 °/µm; VEGF-A: 3.77 °/µm; p = 0.054) were less pronounced.

The same vessel parameters (except Hb content) were also evaluated for whole-mount ear imaging using the same vessel analysis pipeline (AVSA) (Figure 3D). Results for the fill fraction and tortuosity of whole-mounts from ears (edge regions) were consistent with the *in vivo* LSOM data, showing a higher fill fraction in VEGF-A mice (WT: 20.27%; VEGF-A: 25.00%; p = 0.044), and no significant difference in tortuosity (WT: 5.40 °/µm; VEGF-A: 5.15 °/µm; p = 0.631) between mice of both genotypes. The vessel diameter in WT mice was larger than in VEGF-A mice in edge regions (WT: 15.10 µm; VEGF-A: 13.98 µm; p = 0.026), which is contradictory to the LSOM data (1.14-fold larger in VEGF-A mice). At base regions, there was no significant difference between WT and VEGF-A mice in both the fill fraction (p = 0.97) and vessel diameter (p = 0.30).

Statistical analysis of histological data from ear sections was also performed (Figure 4B-C). CD31-stains of VEGF-A transgenic mice showed a significant increase in both the fill fraction (*i.e.,* blood vessel area) and total vessel number in all locations (fill fraction: 2.26-fold, p << 0.001; vessel number: 1.54-fold, p = 0.0017), which is consistent with the LSOM data. A detailed statistical comparison (including mean, standard deviation, and p-value) of the results obtained with the three techniques is shown in Table 1.

### Non-invasive imaging of the dorsal skin in comparison to histological analysis of thin sections

**Figure 5:**
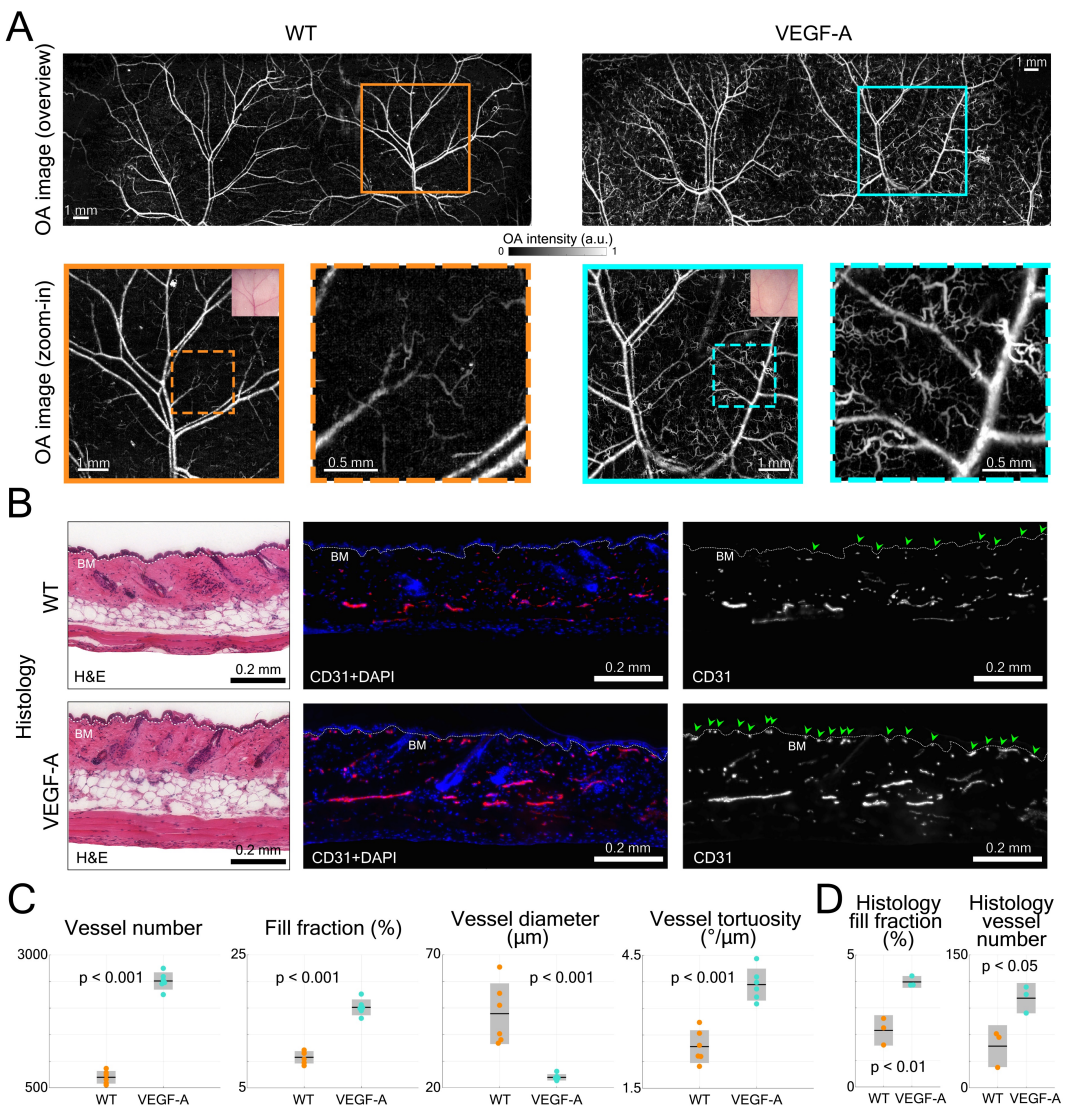
LSOM imaging and histology of dorsal skin vasculature in WT and VEGF-A mice. (**A**) LSOM images of non-wounded dorsal skin showing intricate vascular networks, including arteries, veins, and capillaries. The zoom-in images demonstrate a larger number of tortuous skin microvessels in VEGF-A mice (turquoise dashed box) than in WT mice (orange dashed box). (**B**) Representative photomicrographs of 7 µm H&E and CD31 + DAPI-stained sections of non-wounded dorsal skin from WT and VEGF-A mice. White dotted line represents basement membrane (BM). CD31-positive area is representative for blood vessel area in skin. Nuclei were counterstained with DAPI. Note the increased number of microvessels near the BM in VEGF-A mice (indicated by the green arrows). (**C**) Quantification of vessel number, fill fraction, vessel diameter and vessel tortuosity based on LSOM images. Note that for VEGF-A mice, the bent microvessels largely contributed to a lower average vessel diameter in the dorsal skin. (**D**) Histology quantification of fill fraction and vessel number in non-wounded dorsal skin. CD31^+^ area was corrected by Lyve1^+^ area to account for lymphatic vessels. 2 - 3 regions of interest were analyzed (1000 µm/ROI) per section, n = 3.

The dorsal skin vasculature of WT and VEGF-A mice was next evaluated to visualize and analyze in detail the influence of keratinocyte-specific VEGF-A overexpression. In line with the ear imaging experiment, volumetric LSOM images (1 × 11 × 30 mm^3^ FOV, 20 µm lateral step size) along with smaller dorsal region of interest (ROI, 7 × 7 mm^2^ lateral FOV) at finer 5 µm step size were recorded (Figure 5A). Importantly, no breathing related issues were recognized during our dorsal skin imaging experiments. By employing the 3D-printed dorsal skin mount, only the lower back area was fixed, which allowed the mouse to breath freely without any disturbance generated over the large, imaged area. We also tested and confirmed that the skin mount only applied minimal pressure to flatten the dorsal skin while not perturbing the blood circulation. In both WT and VEGF-A mice, we detected the cutaneous vascular network of dorsal regions, including large artery-vein pairs, smaller interconnected vessels and microvessels (dashed boxes for mice of both genotypes). Remarkably, the number of tortuous skin microvessels was much higher in VEGF-A mice than in WT mice 24. These microvessels were distinctly detected in LSOM images based on the strong intrinsic Hb contrast, whereas only larger blood vessels were visible with the USB microscope (Figure 5A).

The dorsal skin morphology was subsequently evaluated by H&E staining and did not reveal an apparent difference between mice of both genotypes (Figure 5B). In CD31-stained skin sections, more microvessels were observed near the basement membrane (BM) in VEGF-A mice, as indicated by the green arrows (Figure 5B).

Statistical analysis of the LSOM imaging data (*i.e.,* total vessel number, fill fraction, diameter, and tortuosity) was performed to quantitatively compare the difference between WT and VEGF-A mice (Figure 5C). Owing to the large number of tortuous skin microvessels, the total vessel number (WT: 698; VEGF-A: 2509; p-value << 0.001) and fill fraction (WT: 9.6%; VEGF-A: 17.1%; p-value << 0.001) were both significantly higher in VEGF-A mice. The mean vessel diameter in VEGF-A mice (23.9 µm) was strongly dominated by the curly microvessels, compared to WT mice (47.8 µm) with a p-value of 0.00046. Interestingly, the vessel tortuosity in VEGF-A mice was 1.57-fold higher than in WT mice (WT: 2.44 °/µm; VEGF-A: 3.84 °/µm; p << 0.001), while there was no significant difference in the ear skin vessels (Figure 3C).

Quantification of the histological data from non-wounded dorsal skin also showed a significant increase in both the fill fraction (WT: 2.14%; VEGF-A: 3.98%; p = 0.004) and vessel number (WT: 47; VEGF-A: 101; p = 0.022) in the skin of VEGF-A versus WT mice (Figure 5D). A detailed statistical comparison of the data obtained with LSOM and histology is shown in Table 2.

### LSOM and histology detect an increased blood vessel coverage in wounds of VEGF-A mice

**Figure 6:**
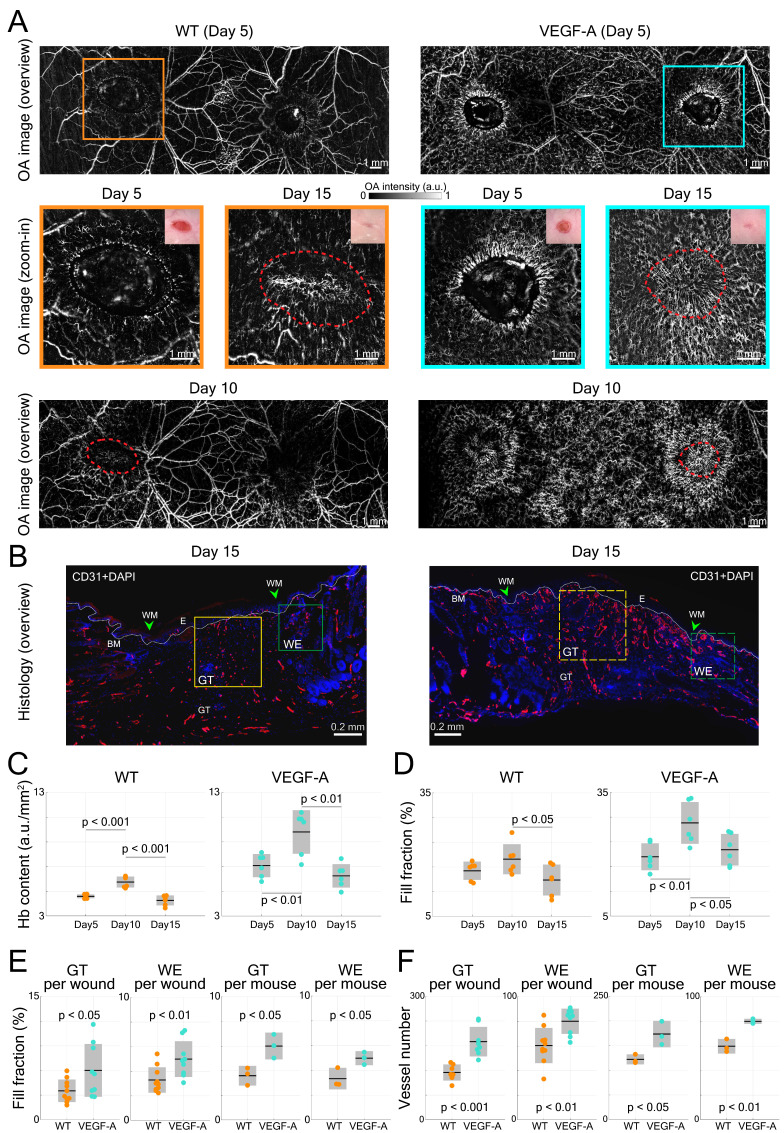
LSOM imaging and histology of wounded dorsal skin. (**A**) Overview LSOM images at day 5 show the tortuous capillary networks forming near the wound border in mice of both genotypes. The zoom-in images at day 5 post wounding highlight the difference in angiogenic sprouts surrounding the wound regions in WT versus VEGF-A mice. The same wounded areas were investigated at day 10 and 15, revealing re-epithelialized wounds at day 10 and mostly re-vascularized wounds at day 15 in mice of both genotypes. The red dashed lines mark the edge of the wounds at day 5 post-wounding. (**B**) Representative photomicrographs of CD31-stained sections from 15-day wounds with boxes showing the area of the late granulation tissue (GT, yellow) and of the wound edge (WE, green) used for analysis. BM: basement membrane (white dotted line), WM: wound margin, E: epidermis. (**C**) and (**D**) Vessel parameters based on LSOM imaging (Hb content and fill fraction) for all wounds over the time course of healing. (**E**) Quantification of the fill fraction of CD31^+^/Lyve1^-^ blood vessel at the wound edge and in the late granulation tissue. Area of blood vessels relative to the GT or WE area per wound and averaged per mouse (3 wounds per mouse, n = 3 mice per group) is shown. (**F**) Quantification of the number of CD31^+^/Lyve1^-^ blood vessels at the wound edge and in the granulation tissue. 3 wounds per mouse, n = 3 mice per group.

VEGF-A plays an essential role in tissue repair-associated vascular cell proliferation, as seen for instance during skin wound healing 28. Therefore, we employed a wound healing model to analyze the angiogenesis in wounds of WT and VEGF-A mice. We first investigated the formation of new blood vessels around the edges of 5-day wounds. This time point represents the peak time of granulation tissue (GT) formation in the wound model that was used in our study. Two GT-filled wounded areas exhibiting a lower signal intensity were observed in the overview LSOM images of WT and VEGF-A mice (Figure 6A). On the other hand, the wound edge (WE) areas were surrounded by neovasculature (*i.e.,* tortuous capillaries), as shown in the zoom-in LSOM images. The GT areas were also clearly observed in the photographs of mice of both genotypes. Noticeably, the distribution of dense microvessels was again significantly higher in VEGF-A mice (day 5, Hb content, WT: 4.6 a.u./mm^2^, VEGF-A: 7.1 a.u./mm^2^, p << 0.001). Progressive wound healing/closure was monitored by LSOM at days 10 and 15 post-wounding (Figure 6A). The WE vascularization had considerably intensified in both WT and VEGF-A mice at day 10 when the wounds were completely closed (re-epithelialized). The Hb content (WT: 5.75 a.u./mm^2^, VEGF-A: 9.80 a.u./mm^2^) and fill fraction (WT: 18.89%, VEGF-A: 27.58%) in the wounds of mice of both genotypes showed the highest values at day 10, while VEGF-A mice manifested elevated values in both metrics (Figure 6C-D). At day 15, all wounds were re-vascularized, and a dense vascular network had formed in the original tissue (red dashed lines). Both vessel parameters declined between day 10 and 15 in both WT and VEGF-A mice, demonstrating a similar pattern of wound closure 28. Statistical analysis of the LSOM data obtained at the different time points of healing is further shown in Table 3.

Interestingly, VEGF-A overexpression not only affected the wound tissue, but also introduced changes to neighboring non-wounded areas. Significant alterations of vascular networks over the healthy dorsal area (*i.e.,* between the wounds) were observed from day 5 to day 10 in the same VEGF-A mouse, while no significant change was observed in the WT mouse (Figure 6A). We quantified the fill fraction of the non-wounded dorsal skin areas in both VEGF-A and WT mice and compared the outcomes at different time points. The fill fraction was increased in both VEGF-A and WT mice from day 0 to day 5 with an average of 6.67% and 7.02%, respectively, showing a largely comparable increase in vasculature in healthy regions adjacent to the wounds. On the other hand, the fill fraction change between day 5 and day 10 was clearly larger for the VEGF-A mice (4.68%) compared to the wild-type mice (0.62%), which is likely attributed to increased VEGF-A levels in the wound tissue.

**Figure 7:**
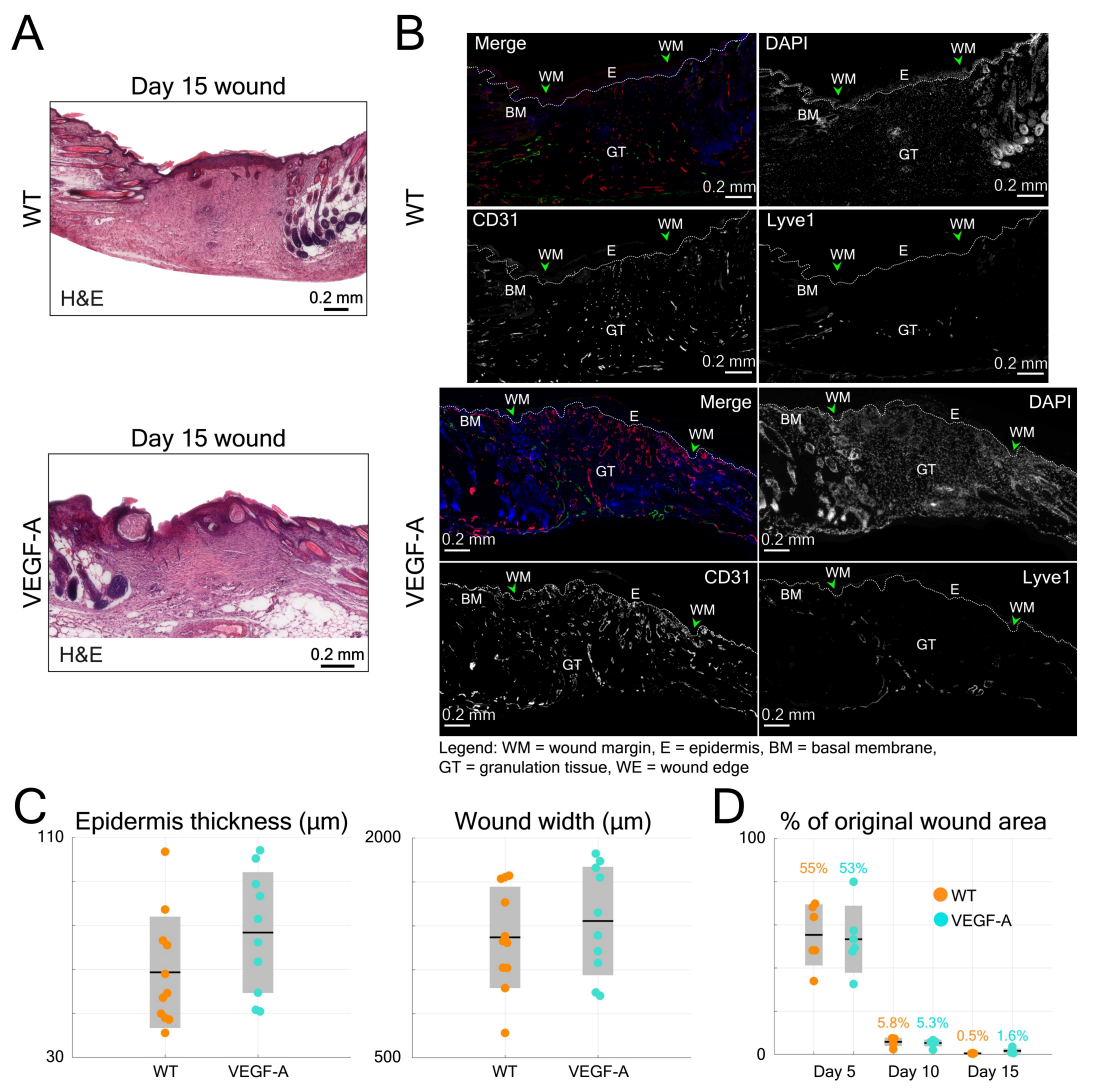
Quantification of histological data from wound sections in WT and VEGF-A mice in comparison to the longitudinal LSOM results. (**A**) Representative photomicrographs of H&E-stained wound sections from day 15 post wounding. (**B**) Additional photomicrographs, showing Lyve1 (Merge, green) and CD31 stainings (Merge, red) and single channel images in wounds of WT and VEGF-A mice. (**C**) Quantification of wound epidermal thickness and wound width in the H&E-stained wound sections. VEGF-A mice show no significant difference in epidermal thickness and wound width. (**D**) Percentile changes of wounded area at different time points as recorded by the *in vivo* LSOM.

Histological analysis of wounds at day 15 post-injury was performed in the same mice (Figure 6B). Blood vessel number and area were evaluated at the WE areas and in the late granulation (early scar) tissue (Figure 6E-F). As shown in the images of both GT and WE (Figure 6B), VEGF-A mice showed a remarkable increase in blood vessel area and number compared to WT mice. This was supported by quantification of the data, showing a significant increase in blood vessel fill fraction (Figure 6E) and number of vessels (Figure 6F), per wound (GT: fill fraction: 1.71-fold, p = 0.039; vessel number: 1.65-fold, p << 0.001; WE: fill fraction: 1.52-fold, p = 0.009; vessel number: 1.33-fold, p = 0.003) and per mouse. These results are in line with the *in vivo* LSOM results where an increase in blood vessel area was observed in the WE and GT areas at this stage. A statistical comparison of the results obtained with WT and VEGF-A mice at day 15 post-wounding is shown in Table 4. Despite the obvious vascular abnormalities, the width and thickness of the wound epidermis were not altered due to the VEGF-A overexpression in 15-day wounds (Figure 7A-C). In addition, the higher number of newly formed vessels in VEGF-A mice had no influence on the wound closure speed, *i.e.,* the wound repair process followed a similar healing pattern, and the wounds were closed at around the same time in mice of both genotypes (Figure 7D).

## Discussion

We employed a recently established non-invasive imaging technology to assess the vasculature in mice with increased expression of a major pro-angiogenic growth factor, both in non-injured skin and during the whole wound healing process. An increase in vascularization due to VEGF-A overexpression can be readily visualized via conventional tissue histology 28. Although being a very useful modality, histological stains are terminal experiments. In contrast, LSOM in combination with AVSA demonstrated the capability to non-invasively observe non-injured skin vasculatures and to monitor development of the skin microvasculature in healing wounds *in vivo*. The results of multiple skin vascular features acquired by LSOM were compared side-by-side with classical histology stainings. In particular, the ear microvasculature was assessed with both LSOM and whole-mount staining to attain a true quantitative comparison between the two techniques by applying the same analysis pipeline. We further included the hemoglobin content and fill fraction measures to rule out any potential bias introduced by AVSA.

The effects of VEGF-A on neovascularization and endothelial sprouting were previously investigated with OA microscopy, yet were limited to the mouse ear 29. In addition, Sun et al. evaluated the effects of microvascular responses to a modified mRNA that encodes VEGF-A, though the skin wounded region was evaluated only by bright-field imaging and oxygen-sensing nanoparticle imaging 30. To the best of our knowledge, the current study is the first to extensively evaluate the effects of skin-specific overexpression of VEGF-A in mouse dorsal skin *in vivo*, including non-invasive quantitative assessment of non-wounded dorsal skin vasculature (Figure 5A) and monitoring of the wound healing progression (Figure 6A). The data showed a similar pattern of angiogenesis as previously shown in our histological and *in vitro* study 28 in both WT and VEGF-A mice. As expected, the VEGF-A mice showed increased blood vessel coverage in both *in vivo* LSOM and histological images. However, the information drawn from classical histology is limited due to inability to infer on the functionality of vessels, especially in healing wounds where new vessels are formed; of which many are non-functional 31. In contrast, the hemoglobin-based contrast retrieved by LSOM directly detects functional vessels, especially microvessels in early wound healing stages. The new approach thus represents a valuable tool for tracking vessel morphogenesis during wound healing or other skin disorders where angiogenesis occurs, reporting concisely on the vascular dynamics and morphology. Additionally, due to the non-invasive and label-free nature of the technique, the mice would still be available for endpoint analysis.

Interestingly, the increased microvascular density of the VEGF-A mice did not speed up the wound healing process. This is in agreement with previous reports showing that VEGF-A is essential for optimal angiogenesis, but not for wound closure 32. This indicates that the angiogenic process in normal wound healing in mice is already optimized, so that additional vascularization does not provide any wound-promoting benefit. In line with this concept, partial inhibition of angiogenesis in healthy mice does not affect wound healing 33,34. It can be also speculated that blood flow may increase due to enhanced vascularization, which might cause more vessel sprouts or enlarged vessels at neighboring places, resulting in better delivery of oxygen and nutrients. However, the newly induced vessels may be immature, and blood flow may not be promoted. This was beyond the scope of this study but should be assessed in the future.

From a technical perspective, scanning of large (centimeter scale) areas with LSOM could be achieved rapidly and smoothly 22. A fast overfly mechanical scanning measure was employed by combining a fast voice-coil stage (VCS), a slow linear stage and the position-based trigger scheme. That is, unlike traditional step-by-step motor scan stage triggered from either laser source or a multipurpose card, the VCS was scanned rapidly and continuously with its position read by a custom-made microcontroller serving as the master trigger source precisely synchronized with laser excitation and data acquisition. Using this approach, we were able to acquire the LSOM images at 12 kHz laser pulse repetition rate without generating vibrations due to rapid acceleration and deceleration of the fast stage. Based on this fast-scanning approach, a quick preview image of 11 × 30 mm^2^ area with 50 µm step size could be acquired within 20 s to find an optimal scanning position, followed by fine-resolution scanning of a smaller ROI with *e.g.,* 20 µm or 5 µm step size (Table 5). On the contrary, imaging of comparable object volumes with fluorescence microscopy techniques using mosaic compounding may take hours to accomplish 35, making their application for large-area *in vivo* skin imaging challenging. Alternatively, an acquisition of microvascular information from the entire mouse ear with whole-mount immunostaining imaging technique is performed post mortem and implies peeling away outer/inner skin for several hours prior to imaging 36. Here, due to the non-invasive nature of LSOM, we were able to perform long-term intravital imaging of the same FOV in the same animal, an important attribute when it comes to an accurate assessment of microvascular morphogenesis in skin wounds.

Based on the optical absorption curve of hemoglobin, the 532 nm wavelength is ideal for generating strong optoacoustic responses from blood 37. It also represents one of the isosbestic wavelengths of oxy- and deoxy-hemoglobin, making it optimal for estimating total hemoglobin concentration of vasculatures with the measurements being independent of saturation 38. Due to the FVB1 genetic background of the mice (white fur), hemoglobin is also the strongest absorbing tissue chromophore in these animals. Therefore, the absorption by melanin is largely averted when employing the 532 nm laser source predominantly generating vascular contrast. Note, however, that LSOM is an optical-resolution microscopy method, hence it would have limited clinical applicability due to inability to focus light through the relatively thick human skin.

The application of the currently proposed AVSA algorithm may result in the skeleton of vessels becoming disconnected, *e.g.,* when a continuous vessel is broken into smaller vessels as false branch points are detected. This issue can potentially be mitigated by processing the images with enough smoothing/filtering during the contrast enhancement procedure or selecting a binarization method with an adjustable sensitivity (*e.g.,* adaptive binarization method), so that false branches are not detected. Alternatively, images from individual cross-sections can be processed so the superficial and deeper vessels are segmented separately, thus averting the issue of small vessels crossing larger underlying vessels in the same image 22.

Whole-mount ear imaging in mice of both genotypes was conducted and evaluated by using the same AVSA to reduce any potential bias on histological analysis, thus facilitating LSOM-based analysis of the vascular metrics. The discrepancy between statistical analysis on the LSOM datasets and whole-mount histological images pertaining vessel diameter in the edge regions (LSOM: 17.7 µm in WT versus 20.1 µm in VEGF-A; whole-mount: 15.1 µm in WT versus 14.0 µm in VEGF-A) can partially be attributed to the different method of volumetric data acquisition from an intact object versus tissue sections with an additional major source of mismatch being the inferior lateral resolution of LSOM. In order to effectively match the curved surface of the mouse head, we had to opt for a large (> 1 mm) depth-of-focus, thus compromising on a relatively large 7.5 µm light spot size. In comparison, the < 2 µm resolution of the whole-mount staining reveals smaller microvascular structures in the ear. Depending on the application and desired FOV, the lateral resolution of LSOM can be further improved by increasing the numerical aperture of the focusing lens, at the expense of diminished depth of focus and/or imaged area. Additionally, CD31-stained whole-mount imaging detects both blood and lymphatic vessels, which may lead to another source of discrepancy. Note that due to the discontinuity of stained vasculature, large diameter vessels were erroneously recognized as many tiny vessels by the AVSA algorithm. Thus, the tortuosity accuracy could be further improved by attempting a more homogenous vessel staining.

Hair regrowth after depilation in both WT and VEGF-A mice adversely affected the LSOM imaging quality in the wound healing experiments. This compromised the optical focusing capabilities due to scattering and acoustic coupling issues, further diminishing the signal-to-noise ratio and effective imaging depth. In the future, the skin hair growth cycle needs to be carefully monitored for rendering consistent LSOM images throughout the wound healing process. Alternatively, SKH1 hairless mice could be used for long-term skin vasculature monitoring.

Direct physical contact of coupling medium with the skin wound may lead to an increased risk of infection. Indeed, non-contact imaging techniques, such as optical coherence angiography, largely decrease the risk of infection for wound monitoring. Proof-of-concepts of such non-contact studies have recently been reported 39,40, albeit the FOV and overall image quality were inferior to contact-based imaging methods. In the current study, we followed strict hygiene procedures for preventing the potential infection due to the contact of wound areas with coupling medium, thereby avoiding skin infection.

It should be also noted that classical histology of dorsal skin and wounds is based on side-view tissue sections, whereas the LSOM images represent planar projections of volumetric data along the depth dimension, *i.e.,* top views. While LSOM images generally provide a more comprehensive undisrupted view of the vascular network, the inferior axial resolution, which is limited by the available detection bandwidth of the ultrasound transducer, compromises the depth resolving capacity of the method. Thus, optical microscopy could be used to complement the corresponding high-resolution information along the depth direction.

Spectroscopic optoacoustic imaging techniques employing several excitation wavelengths are generally capable of providing oxygen saturation readings to assess the relation between angiogenesis and oxygen metabolism 15,41. Additional illumination wavelengths could then be integrated into LSOM to provide additional functional information on the wound healing process. Blood flow is yet another major parameter for evaluating skin vascular diseases, which can be retrieved via a variety of spatial or temporal modulation techniques 42. Also, it was recently demonstrated that blood flow can be quantified by evaluating the flow-induced temporal decorrelation of optoacoustic signals recorded from mouse ear vasculatures 43.

In general, optoacoustic methods can be employed for skin research in both preclinical and clinical settings to diagnose various diseases such as psoriasis, scleroderma, and skin cancers 15,21,44. It should be emphasized that optical absorption contrast is not solely restricted to examination of blood-related metrics and vascular malformations, but can also map the distribution of other molecules such as melanin, lipids, collagen and water 45, thus aid the detection of melanoma 15 or characterization of the entire pilosebaceous unit 46.

On the other hand, the lymphatic system, present in most organs of the body, is linked to the development of many different pathologies, such as lymphedema and chronic inflammation 47. Therefore, visualization of the lymphatic vascular system, *i.e.,* the blind-ended lymphatic vessels/capillaries, may further help to optimize therapeutic interventions in lymphatic disorders 28,47. Previously, optoacoustic tomography approaches providing spatial resolution in the 150 µm range have been used to investigate the lymphatic vascular network in both limbs of healthy volunteers and lymphedema patients, with the help of optical tracer indocyanine green (ICG) 47. That is, by using commercial optical tracers or tailor-designed markers of lymphatic vessels, the LSOM approach can potentially be used to simultaneously identify both blood and lymphatic vasculature with high spatial resolution 28,47.

In summary, we successfully demonstrate the capability of LSOM for non-invasive longitudinal monitoring of skin vascularization in transgenic VEGF-A mice *in vivo*. The epi-illumination design of LSOM could be further employed for examinations of *ex vivo* skin samples, and even monitoring of skull/brain regions in murine models 19,20. The results show the capability of LSOM for quantitative longitudinal *in vivo* evaluation to replace or supplement the cumbersome *ex vivo* histological procedures, thus demonstrating its broad potential for skin research and interrogation of diseases. Future work will focus on non-invasive assessment of therapeutic interventions targeting skin tumors and other diseases using LSOM.

## Materials and methods

### Large-scale optoacoustic microscopy (LSOM) setup

Microvascular networks of mouse skin were evaluated by the label-free LSOM imaging technique (Figure 1A), based on an intrinsic optical absorption contrast of living mammalian tissues primarily stemming from light absorption by hemoglobin. For volumetric imaging, a fast voice coil stage (VCS, X-DMQ12P-DE52, Zaber Technologies, Canada) and a slow linear stage (LNR50SEK1/M, Thorlabs, Germany) were integrated to scan the imaging target with high-speed in a sinusoidal pattern. Note that a custom-made, microcontroller-based read-out board (Teensy 3.6, PJRC, USA) was employed to read the position of the fast VCS in real time, allowing a position-based trigger scheme for a smooth LSOM image acquisition. The scanned area can be selected according to the region of interest with the scan speed determined by the desired spatial resolution. For instance, coarse-resolution overview images covering the entire ear were acquired with 20 µm step size (*e.g.,* 10 × 10 mm² lateral FOV), while zoom-in images close to the ear edge (*e.g.,* 1.7 × 1.7 mm² FOV) were recorded with a finer 5 µm step size. The scan parameters of LSOM images and their corresponding scan time are listed in Table 5.

A 532 nm nanosecond laser (Onda, Bright Solutions, Italy) was used to generate laser pulses, while the energy was tuned by a pair of half-wave plate and polarizing beam splitter. Directly attached to a single-mode optical fiber, the custom-designed gradient index (GRIN) lens (Grintech, Germany) was used to focus the light onto the target (*i.e.,* hemoglobin in perfused skin blood vessels) with a per-pulse energy < 900 nJ and a repetition rate up to 12 kHz. The generated broadband US signals of vessels were recorded by a spherically focused polyvinylidene fluoride (PVDF) US sensor (35 MHz central frequency, > 100% bandwidth, Precision Acoustics, UK), which was coaxially and confocally aligned with the GRIN lens. The signals were then amplified by a 24 dB low-noise amplifier (ZFL-500LN, Mini-Circuits, USA) before being digitized by a 2-channel, 250 MS/s, 16-bit data acquisition (DAQ) card (M4i4420-x8, Spectrum, Germany). Afterwards, the recorded signals were reconstructed and analyzed using our developed algorithms in Matlab. Due to the low numerical aperture (NA = 0.05) of the GRIN lens, a 7.5 µm spot size with an extended depth of focus > 1 mm was achieved, thus averting the need for optical sectioning and sequential acquisition of images from different depths 22.

### Automatic vessel segmentation and analysis algorithm (AVSA) for skin vessels

A previously described automatic vessel segmentation and analysis algorithm (AVSA) 22,48,49 was optimized to quantify the relevant metrics of skin microvascular networks for all the acquired LSOM and whole-mount experimental datasets in this study. All vessels underwent the same vessel analysis pipeline (Figure 2A) to calculate the vessel parameters. The volumetric LSOM datasets were first converted into 2D images by calculating maximum intensity projections (MIP) along the depth dimension. Contrast limited adaptive histogram equalization (CLAHE) and filtering were then applied to the MIP datasets for adjusting the dynamic range and smoothing the images, respectively. In the next step, Otsu's algorithm was employed to perform image thresholding and binarization 50, followed by the vessel skeletonization procedure. In the end, branch points, center lines and edges of blood vessels were identified, and vessel parameters (*e.g.,* vessel angle for tortuosity) were calculated for further statistical analysis of the vascular network (Figure 2B).

The following parameters were analyzed for a quantitative comparison between WT and VEGF-A mice: *Hemoglobin (Hb) content* was calculated based on the sum of raw LSOM image intensity values divided by the total imaged area, aiming to rule out any potential bias introduced by the CLAHE and filtering procedures. *Fill fraction* - the total blood vessel area divided by the total imaged area - is the most straightforward metric to evaluate the skin vasculature distribution. *Number of identified vessels*, *vessel diameter* and *vessel tortuosity* (sum of all angles divided by vessel length) were also included into the assessment. The median values of the vessel parameters, either per location or per mouse, were extracted with the mean values ± standard deviations calculated and presented for the LSOM image statistics. The p-value was calculated using an unpaired two-tailed t-test, where p-value < 0.05 was considered as statistically significant and p-value < 0.001 as statistically highly significant. The total time for processing one MIP image was less than 40 s.

### Animals and skin experiments

K14-VEGF-A mice, which express *VEGF-A* under control of the keratin 14 promoter (FVB1 genetic background) in basal keratinocytes of the skin and other stratified epithelia and in the outer root sheath of the hair follicles, were evaluated for their alterations in the skin vasculature in comparison to their WT littermates 24. Mice were housed at 12-hour-light/dark cycle, 20 - 24 °C temperature, and 50 - 70% humidity under specific pathogen-free conditions and received food and water *ad libitum*. Mouse maintenance and all animal experiments of this study were approved by the local veterinary authority (Kantonales Veterinäramt Zürich, Switzerland).

For* in vivo* skin imaging, mice were anesthetized using a mixture of isoflurane and oxygen/air (20/80%) for all experiments. The animals were placed on a self-regulated heating pad at 37 °C (Figure 1C) to maintain their body temperature during the entire experimental procedures. For ear imaging, the mouse ear was placed on a customized 3D-printed board. To ensure a good acoustic coupling between the US sensor and ear, a small amount of coupling medium (70% US gel and 30% phosphate-buffered saline) was applied to the mouse skin. Then, a customized thin film holder filled with water was placed on top of the coupling medium allowing for LSOM image acquisition.

A full-thickness excisional wound model was used in this study 51. Female mice (9 weeks old) were anesthetized with their backs shaved and hair removed and cleaned as previously described 22. Each mouse carried a total of 4 dorsal full‐thickness excisional wounds (5 mm in diameter) generated with disposable/surgical biopsy punches (Figure 1B). Wounds were allowed to heal without dressing 28. The wounded dorsal areas were imaged by LSOM at day 5, 10 and 15 post-wounding (n = 3 per group). Non-wounded mice were also included for supporting histology, LSOM controls and ear whole-mount staining. Fifteen days post-wounding, wounds were prepared for histology/immunostaining. They were either fixed in AcEtOH (1% acetic acid in 95% EtOH) and then embedded in paraffin or frozen in tissue freezing medium (Leica Biosystems, Wetzlar, Germany). Wounds were bisected along the cranial-caudal direction and embedded, thus sections were always aligned along the same axis and represented the central portion of the wound.

### Histological analyses of the ear and dorsal skin

For H&E staining, 7 µm sections were deparaffinized and stained. For immunofluorescence staining, 7 µm sections were blocked with donkey serum (Sigma-Aldrich) and incubated overnight with goat biotinylated anti-Lyve1 (1 : 50, R&D) and rat anti-CD31 (1 : 20, Dianova, Hamburg, Germany) primary antibodies, followed by donkey anti-rat AlexaFluor 488 and anti-biotin streptavidin AlexaFluor 594 secondary antibodies (1 : 200, Thermo Fisher Scientific, Waltham, MA), and counterstained with Hoechst 33342 (Thermo Fisher Scientific).

For immunofluorescence staining of frozen skin samples, 7 µm cryosections were fixed in cold acetone (5 min) and cold methanol (70%, 2 min), incubated overnight with rabbit anti-Lyve1 (1 : 600, Angiobio, San Diego, CA) and rat anti-CD31 (1 : 200, Becton Dickinson, Franklin Lakes, NJ) primary antibodies, followed by anti-rat AlexaFluor 488 and anti-rabbit AlexaFluor 594 secondary antibodies (Thermo Fisher Scientific), and counterstained with Hoechst 33342.

For ear whole-mount immunofluorescence staining, the ears were split and the cartilage was gently removed. Tissue was fixed with 4% PFA for 2 h at 4 °C and washed with PBS/0.3%Triton X-100. Subsequently, ear skin sections were blocked for 2 h in washing solution containing 0.3% bovine serum albumin and 5% donkey serum and incubated overnight with rabbit anti-Lyve1 (1 : 500, Angiobio) and rat anti-CD31 (1 : 200, Becton Dickinson) primary antibodies, followed by 2 h washing and 4 h incubation with anti-rabbit AlexaFluor 488 and anti-rat AlexaFluor 594 secondary antibodies (1 : 300, Thermo Fisher Scientific). Nuclei were counterstained with Hoechst 33342.

### Image acquisition and analysis of histological skin sections

Light microscopy and fluorescence images were acquired with a Pannoramic 250 Slide Scanner (3D Histech, Budapest, Hungary) at 20× magnification. Whole-mount ear images were acquired with a Leica TCS SP8 inverted system at 10× magnification. All histological sections were analyzed in Fiji 52. For H&E stains, wound margins were defined by the presence of skin appendages. For epidermal thickness, the whole epidermis area covering the granulation tissue was identified and divided by the traced length of the basement membrane. For wound width, the distance between the first hair follicles on both sides of the wound was determined. For Lyve1/CD31-stained sections and blood vessel quantification, wound margins were identified as described above. The wound edge was measured as the area 500 µm outwards of the wound margin away from the granulation tissue. The granulation tissue was defined as the granular appearing tissue between the two wound margins. Single-channel 8-bit raw images of CD31 were carefully thresholded and vessel-like structures manually filled and corrected for overexposure. Subsequently, CD31^+^ vessels were controlled for lymphatic vessels by immunostaining for the lymphatic endothelial cell marker Lyve1^+^. In non-wounded dorsal skin sections, the CD31-positive area was quantified in 1 cm wide representative areas and corrected by the Lyve1-positive area for lymphatic vessels. Ear sections were analyzed at the base, base-middle, middle-tip and tip in a 750 µm wide representative region on the dorsal side. Vessel-like structures were filled, and area fraction was measured per pre-defined ROI and the number of vessels counted.

## Author contributions

JR, LB, SW, MD and DR conceived the study. JR and YL conducted the longitudinal LSOM experiments. LB and MG conducted ear whole-mounts and classical skin histology imaging. JR implemented the vessel analysis algorithm. LB and YL performed vessel segmentation and analysis. LB and YL prepared the figures. YL prepared the tables. SW, MD and DR supervised the study. All authors contributed to writing and revising the manuscript.

## Figures and Tables

**Table 1:** Comparison of ear skin vessel parameters quantified by the three techniques

Vessel parameters	*In vivo* LSOM			Whole-mount (edge)			Histology (per mouse)		
	WT	VEGF-A	p-value	WT	VEGF-A	p-value	WT	VEGF-A	p-value
Hb content (a.u./mm^2^)	9.2±2.3	15.2±2.7	2.8×10^-4^	-	-	-	-	-	-
Fill fraction (%)	11.1±2.2	20.4±4.0	4.9×10^-5^	20.3±2.2	25.0±2.3	0.044	6.9±2.4	15.6±3.1	1.2×10^-6^
Diameter (µm)	17.7±2.2	20.1±2.0	0.038	15.1±0.5	14.0±0.4	0.026	-	-	-
Tortuosity (°/µm)	3.1±0.6	3.8±0.7	0.054	5.4±0.8	5.2±0.5	0.631	-	-	-
Number	-	-	-	-	-	-	36±12	55±11	0.002

**Table 2:** Comparison of dorsal skin vessel parameters retrieved by *in vivo* LSOM and histology

Vessel parameters	*In vivo* LSOM			Histology (per mouse)		
	WT	VEGF-A	p-value	WT	VEGF-A	p-value
Number	698±120	2509±164	8.8×10^-10^	47±23	101±17	0.022
Fill fraction (%)	9.6±0.9	17.1±1.2	2.5×10^-7^	2.1±0.6	4.0±0.2	0.004
Diameter (µm)	47.8±11.4	23.9±1.2	4.6×10^-4^	-	-	-
Tortuosity (°/µm)	2.4±0.4	3.8±0.4	5.8×10^-5^	-	-	-

**Table 3:** Statistical skin vessel parameters extracted from the LSOM images during wound healing

Vessel parameters	Day 5 post-wound			Day 10 post-wound			Day 15 post-wound		
	WT	VEGF-A	p-value	WT	VEGF-A	p-value	WT	VEGF-A	p-value
Hb content (a.u./mm^2^)	4.6±0.2	7.1±0.9	7.6×10^-5^	5.8±0.5	9.8±1.8	2.8×10^-4^	4.3±0.4	6.3±1.0	8.0×10^-4^
Fill fraction (%)	16.1±2.2	19.4±3.3	0.067	18.9±3.7	27.6±5.1	0.007	13.8±3.8	21.1±3.8	0.008

**Table 4:** Statistical skin vessel parameters in histology at Day 15 post wounding

Vessel parameters	GT (per wound)			WE (per wound)		
	WT	VEGF-A	p-value	WT	VEGF-A	p-value
Fill fraction (%)	3.5±1.4	6.0±3.2	0.039	3.3±1.0	5.0±1.5	0.009
Number	115±20	190±36	2.9×10^-4^	60±14	80±10	0.003
	**GT (per mouse)**			**WE (per mouse)**		
	WT	VEGF-A	p-value	WT	VEGF-A	p-value
Fill fraction (%)	3.6±0.8	6.0±1.1	0.025	3.3±0.9	5.0±0.6	0.036
Number	122±10	174±27	0.025	60±6	80±2	0.003

**Table 5:** *In vivo* LSOM scan parameter overview

Usage or figure panel	Scan size (mm × mm)	Step size (µm)	Scan time (min:sec)
Preview scans^1^	11 × 30	50	0:21
Fig. 5A^2^, Fig. 6A^2^	11 × 30	20	1:53
Fig. 3A^2^	10 × 10	20	0:34
Fig. 3A^3^, Fig. 5A^3^, Fig. 6A^3^	7 × 7	5	4:35

^1^The pulse repetition rate of the preview scans was 10 KHz, and the repetition rate for other image acquisitions was 12 KHz. ^2^Overview images.^ 3^Zoom-in images.
